# Supplementary material for: Are carbon emissions trading and green financial instruments synergistic? -Comprehensive quantitative research based on content analysis
Source: PLoS One. 2024 Mar 7;19(3):e0298601. doi: 10.1371/journal.pone.0298601 (PMC10919601; doi:10.1371/journal.pone.0298601)
Supplement: S1 Data — (ZIP) [file pone.0298601.s001.zip › Data/地级市Z_F工作报告.zip!/┴╔─■/░░╔╜╩╨/└┤╫╘╣1⁄2╓┌║┼í╛┬φ┐╦╩2╛▌═°í┐_2003.docx]

各位代表：

现在，我代表市十二届人民政府向大会报告工作，请予审议，并请政协各位委员和列席人员提出意见和建议。

一、五年来政府工作回顾

市十二届人民政府任期从1998年1月至今已满五年。五年来，在省委、省政府和市委的领导下，市政府高举邓小平理论伟大旗帜，深入实践“三个代表”重要思想，全面贯彻党的十五大和十六大精神，深化改革，扩大开放，促进了国民经济和社会事业健康发展，保持了社会稳定。特别是进入新世纪，围绕实现高起点开局和跨越式发展，通过深入开展“塑造鞍山新形象”大讨论活动，增强忧患意识，理清发展思路，确立了建设现代工业强市、优秀旅游名市和先进文化城市的发展目标，确定了以开放促改革、促调整、促发展，全面建设开放型地区经济的战略构想，树立了“经营城市”和建设“绿色鞍山”的理念，提出了“谋划大事、自我加压、破解难题、深度推进”的创新工作要求，推动鞍山经济步入加速发展的快车道。预计2002年全市实现国内生产总值709亿元，比上年增长11%，五年平均增长10.1%。其中，农业增加值52亿元，年均增长9%；工业增加值349.7亿元，年均增长9.4%；第三产业增加值266亿元，年均增长11.3%。实现地方财政收入32.6亿元，比上年增长14.1%，五年平均增长11%。县区财政收入均超亿元。累计完成全社会固定资产投资599亿元，比前五年增长42.4%。城市人均可支配收入6800元，五年增长34.4%；农民人均纯收入3780元，五年增长20%。

（一）扩大开放，规划和引进一批有牵动力的大项目，增强了经济发展后劲。五年实际利用外商直接投资5亿美元，比前五年增长2.2倍，新办“三资”企业437家。积极争取项目争取资金，实际到位资金以年均25%的速度递增。通过举办中国·鞍山（北京）推介会、海外学子创业周等一系列招商活动，引进了台湾纺织工业园、韩国大邱纺织工业园等一批重大外资项目，美国欧文斯·科宁、加拿大好运集团、台湾中橡、香港华润、青岛啤酒等一批知名企业来鞍投资。与香港中旅集团、青岛海尔集团、清华大学、北京大学、上海交大建立了长期合作关系。与韩国安山、德国埃森等14个国外城市和无锡、九江等一批国内城市建立了友好合作关系。坚持以项目为核心，市地方规划投资亿元以上项目100个，投资总额442亿元。其中，5亿元以上项目17个，投资额290亿元；工业项目53个，投资额305亿元；已开工建设45项，总投资91.4亿元。

（二）深化改革，加快产业结构和所有制结构调整，提高了经济运行质量。全面落实党在农村的各项政策，在稳定家庭联产承包责任制的基础上，深化土地流转制度改革，顺利开展了撤乡并镇和区划调整工作。大力推进农业产业化经营，发展生态农业、设施农业、订单农业和创汇农业，形成了优质米、蔬菜、食用菌等十大主导产品，建设了6个农业科技示范园区，千山宁远、海城王家堡子等4个现代农业园区进入省级农业园区行列。培育了364个龙头企业，其中省市级龙头企业14个，龙头企业带动农户比例达到45%，促进了传统农业向现代农业转变。完成退耕还林和荒山造林17万亩，植树造林合格面积68万亩。对辽河、浑河、太子河鞍山段进行综合整治，防洪标准由20年一遇提高到50年一遇。在巩固国有大中型企业三年改革与脱困成果基础上，积极推进国有经济布局调整和企业公司制改造，组建了冶化建、机电、轻纺、物资、建工等5个资产经营公司，完成了地方300户国有中小企业改制任务，在19户国有大中型企业建立了现代企业制度。总结推广“两啤一化”模式，对21户地方国有大中型企业实施了脱胎换骨式改造。全市工业经济综合效益指数达到107.6%。通过政策引导、放手发展、积极扶持，西洋耐火、宝德轧钢等一批民营企业不断壮大，民营经济占全市GDP的比重达到48.3%。加大旅游资源开发和宣传力度，千山和玉佛苑晋升为国家4A级旅游景区（点），千山仙人台景区被认定为国家级森林公园。成功举办了三届中国·鞍山千山国际旅游节和首届中国·鞍山玉佛文化节，旅游综合收入五年平均增长23%。现代流通业态方兴未艾，各类超市和连锁店发展到320家。

（三）科技创新，大力发展高新技术产业，高新区实现了历史性跨越。鞍山被确定为国家技术创新试点城市和全国制造业信息化工程重点城市。五年累计取得市级以上科研成果364项，获得国家、省（部）级科技进步奖60项，申报专利1910项。培育发展省级高新技术企业119家。科技进步对工业经济的贡献率达到40％以上，对农业经济的贡献率达到55%以上。高新区实行封闭式管理，调整了总体规划，减少居住用地，增加产业项目用地。对进区企业和项目免缴各种行政事业性收费，成为“无费区”。先后建成了海外学子创业园、鞍山软件园和科大科技园，引进了托普软件、清华紫光等一批高科技企业。创业服务中心晋升为国家级，累计孵化企业286家。高新区五年累计完成基础设施建设投资4.6亿元，建成标准厂房1.4万平方米。引进项目912个，其中投资亿元以上项目10个，实际利用外资5123万美元。2002年高新区完成技工贸总收入150亿元，五年平均增长108%；实现高新技术产品产值50亿元，五年平均增长50%；实现全口径财政收入3亿元，五年平均增长42%。高新区整体发展水平和对外形象有了显著变化，在国家级高新区的排位由51位上升到36位。

（四）经营城市，全面改善经济发展软硬环境，塑造了现代文明、整洁优美的城市形象。通过土地出让、市场化运作和适当融资等方式，五年累计完成城建投资40亿元。建成了以五一路立交桥为轴心的“十”字型城市交通主骨架，改造和大修51条主次干道，46处马路市场实现了退路进厅，畅通工程达到国家A类一等模范城市标准。实施了全日制供水工程和城乡电网改造工程。新建了二一九广场，对胜利、站前和人民公园等9个城市广场进行了高标准美化、绿化、亮化，为广大市民提供了休闲娱乐的高品位场地。城市植树537万株，新增绿地1129万平方米，城区绿化覆盖率达到35％。新建和改造居民小区71个，基本完成棚户区改造，建设住宅500万平方米。对建成区临时和违章建筑进行了全面拆扒，完成了市区主街主路照明设施更新和楼宇粉饰。西部第二污水处理厂已完成一期工程，实施了120项环境污染治理工程，城市空气质量全年有一半以上天数达到国家二级标准，太子河鞍山段主要污染物治理后水质达到国家地面水五类标准。规划建设了8个省级以上小城镇示范点，改造了鞍海路、鞍腾路、鞍羊路等干线路网，提前三年实现了乡乡通油路和村村通公路。适应入世要求，开展了软环境建设绿色行动，出台了一系列规范软环境建设的政策规定，压缩行政审批事项83.5%。在省内率先成立了市县两级公共行政服务中心以及经济发展软环境投诉中心、经济政策查询中心和外商投资企业代理服务中心，为海内外投资者、企事业单位和广大市民提供了便捷高效的服务，增强了城市的吸引力和竞争力。

（五）地企融合，积极为在鞍中省直企业创造良好环境，开创了地方与鞍钢联合协作的新局面。树立“地企融合、共兴共荣”理念，积极为中省直企业改革发展提供服务，全面接收了中省直企业所属中小学校，实现了中省直企业职工失业保险属地化管理。鞍钢形成了母子公司体制，建立了现代企业制度。实施了1780、1700、钢轨万能轧制生产线等一系列重大技术改造，主体装备达到世界先进水平，钢铁产量双超1000万吨，生产经营跨越式发展，对市本级财政贡献率始终保持在40%以上。地方和鞍钢分别成立了联合协作工作机构，建立了重大问题及时协商制度。按照市场经济规律，采取资本运作方式，鞍钢出资5000万元一次性买断了闲置八年之久的东北风厂房场地，地方帮助协调化解各种债务2.2亿元，建设了年产30万吨的两条彩色涂层钢板生产线。帮助解决鞍钢与冀东水泥合资生产150万吨水泥熟料项目建设中遇到的各种困难，实现了企业在地方注册。支持鞍钢铸管厂搬迁改造，将3000万元土地补偿费返还给鞍钢，建成了年产8万吨的两条球墨铸铁管生产线，同时也极大地改善了城市环境。通过这三个示范项目，探索出地方与鞍钢联合协作的多种成功模式，为今后工作奠定了坚实基础。

（六）促进就业，积极推进社会保障体系建设，保持了社会稳定。认真组织建立完善的社会保障体系试点工作，社会养老、失业、医疗保险覆盖面分别达到100%、99%和75%。五年地方财政用于社会保障支出6.5亿元，累计发放下岗职工基本生活费4.5亿元，支出养老金71.4亿元。全部关闭了企业再就业服务中心，有12.8万人与企业解除了劳动关系，对失业并轨人员做到了接续养老保险和失业保险。健全和完善了劳动者自主择业、市场调节就业、政府促进就业的新机制，开展就业培训和就业援助，兴办再创业企业，创办社区保安、保洁、商品配送和家政服务四大公司，走出了一条依托社区的多渠道就业之路。五年累计安置就业34万人次，涌现出小铅笔编织社、妈咪贝贝月嫂服务中心和康泰华刮痧中心等一批再创业企业典型。全面落实城镇居民最低生活保障制度，建立了科学的组织体系和规范的审核发放体系，累计发放最低生活保障金1.2亿元，做到了应保尽保。积极开展扶贫救助活动，发动社会力量筹集款物3亿元，累计救助150万人次，为516户农村贫困户和1594户城市困难居民解决了住房问题。有效化解金融风险，“两债”余额下降80%。加强安全生产和安全防火工作，杜绝了重大安全事故发生。深入开展严打专项整治斗争，坚决取缔“法轮功”等邪教组织，强化社会治安综合治理，有效遏制了重特大刑事案件的发生。

（七）“四城”同创，全面加强精神文明建设，推动了社会事业全面进步。全市动员，全民参与，认真组织“创城”活动，进入中国优秀旅游城市行列，荣获创建全国文明城市工作先进城市称号，成为东北地区第二个国家卫生城市，蝉联全国双拥模范城。贯彻落实《公民道德建设实施纲要》，提高了全市人民的思想道德素质和城乡整体文明程度。巩固“普九”成果，大力推进了素质教育。强化高中教育和职业教育，高中阶段入学率和高考升学率分别提高13个百分点和36个百分点。基本完成了鞍山科技大学升级换建和鞍山师范学院重组整合。恢复了市新华书店等主要文化设施的基本功能，建成了市少儿阅览中心，组建了市艺术学校。深化专业表演艺术团体改革，创作出一批优秀剧目。创建大型文化广场10处，发展各级各类文化网点296个，丰富了群众文化生活。实施区域卫生规划，建立了新的卫生监督和疾病预防控制体系。加强了农村医院建设。广泛开展城市社区卫生服务，可服务人口达到90%，主要健康指标达到国家先进水平。建成了市体育中心，成功承办了辽宁省第九届运动会，实现了参赛成绩进入省内前三名、办会接待水平超过前八届、商业化运作筹措办会资金“三个既定目标”，凝炼出“万众一心、敬业奉献、追求卓越、铸就辉煌”的九运精神。民族、宗教、侨务政策得到全面落实。对台经贸工作成效显著，台商对鞍山的认知度明显提高。民兵预备役建设不断加强。人防工作成为全省一面旗帜。军政军民关系进一步发展。广播电视宽

带网接入20万户。气象服务和接待服务达到省内一流水平。老龄事业在实践中得到创新。完成了建国后鞍山首届修志工作。档案工作较好地发挥了服务经济和服务社会的作用。人口与计划生育、妇女儿童和地震工作进入全国先进行列。

（八）依法治市，不断加强政府自身建设，改进了机关工作作风。认真组织“三五”、“四五”普法活动，有效实施了法律援助。加强地方行政立法工作，提请人大审议地方性法规16件，颁布实施行政规章60件。清理法规、规章243件，废止政府文件46件。建立行政执法责任制，聘请政府法律顾问，规范行政执法行为，行政复议胜诉率提高20个百分点。坚持重大事项及时向人大及其常委会报告和向人民政协通报，自觉接受人大依法监督、政协民主监督和人民群众社会监督，人大代表建议和政协委员提案办结率100%。完成市县两级政府机构改革，市级政府机构精简28.6%，压缩行政编制31.3%。加大反腐倡廉力度，提高了公务员素质。采取《政府工作任务书》等有效形式，使政府各部门自我加压，开创了工作新局面，树立起廉洁高效的政府形象。加强信访和市民投诉受理工作，创办《今晚会见》栏目，设立政府网站和市长信箱，密切了与人民群众的血肉联系，营造出奋发向上、积极进取的社会氛围。

各位代表，过去五年改革开放和现代化建设所取得的成就，既为十二届政府工作画上了一个圆满句号，更为我们在十六大精神指引下全面建设小康社会奠定了坚实基础。这是全市人民努力拼搏的结果，也是社会各界大力支持的结果。在此，我代表政府全体成员，向全市广大工人、农民和知识分子，向鞍钢等中省直单位和市属企事业单位、驻鞍部队、武警官兵、公安干警、大中小学校师生、离退休干部、广大公务员以及所有关心和支持鞍山发展的海内外朋友，表示衷心的感谢和崇高的敬意！

在肯定成绩的同时，我们也清醒地看到，在前进的道路上还面临着许多困难和问题。经济结构的战略性调整还不到位，产业结构还处于较低层次，新的经济增长点不够突出；市场经济体制还不完善，国有企业改革与发展的任务仍很艰巨；对外开放的深度和广度与经济发展的要求还存在较大差距，招商引资的大项目不多，投资环境有待于进一步改善；城乡居民收入增长缓慢，下岗失业人员较多，部分群众生活仍很困难，就业和社会保障的压力较大；政府机关行政效能不高、创新突破意识不强、工作作风不够扎实等问题还比较突出，个别干部还存在腐败现象。对这些问题，我们都要予以重视，并在今后工作中采取有效措施，认真加以解决。

各位代表，十二届政府任期的五年是不平凡的五年，我们经历了亚洲金融风险的考验，经历了国有企业三年改革与脱困的磨练，经历了中国入世的挑战。五年的实践，使我们加深了对市情的认识，进一步理清了未来发展的思路，在组织经济和社会发展方面也积累了一些经验。我们深深感到，开放是鞍山发展的根本出路，开放的力度决定改革的深度、调整的进度和发展的速度；项目是经济工作的核心，只有源源不断地实施大项目，才能实现经济的跨越式发展；投资环境是提升城市综合竞争力的核心要素，只有坚持不懈地改善投资环境，才能不断增强城市的吸引力和凝聚力；人民群众是鞍山的建设者和受益者，只有集中民智，凝聚民力，顺应民心，反映民意，才能真正形成加速发展的强大合力；创新是加速发展和实现赶超的制胜法宝，只有不断地解放思想，战胜自我，超越自我，永远奋斗，才能使鞍山越来越美好！

二、新一届政府工作的指导思想和奋斗目标

新一届政府任期的五年，是全面建设小康社会的关键时期。历史和现实要求我们必须牢牢抓住本世纪前二十年这一重要战略机遇期，集全民之智，举全市之力，以更高的追求，更大的气魄，谱写社会主义现代化建设的新篇章。根据市委九届六次全会做出的战略部署，今后五年乃至到2010年政府工作总的指导思想是：高举邓小平理论伟大旗帜，全面实践“三个代表”重要思想，深入贯彻十六大精神，围绕建设现代工业强市、优秀旅游名市和先进文化城市，继续坚持以开放促改革、促调整、促发展，建设开放型地区经济的战略构想，突出项目核心，强化招商引资，加速经济结构战略性调整，全面扩大社会就业，积极推进科教兴市，提升国民经济整体素质和人口素质，增强可持续发展能力，走上生产发展、生活富裕、生态良好的文明发展道路，为鞍山提前全面建成小康社会、在省内第一批基本实现现代化奠定基础。

今后五年乃至到2010年全市经济和社会发展的主要奋斗目标：

——基本形成靠拢沈大的经济实力。国内生产总值年均增长12%以上，全面完成“十五”计划，到2005年突破千亿元大关，到2007年比2000年翻一番，到2010年人均国内生产总值超过5000美元。地方财政收入年均增长13%以上，到2007年地方可支配财力达到75亿元，到2010年超过100亿元。城市综合竞争力显著增强，跻身省内第一集团。

——基本形成现代大都市的城市框架。以规划建设鞍山新区为切入点，实施鞍山城区南扩、海城北靠、上下合力、共建鞍海新经济带战略，到2010年城市化水平达到60%以上。建成高速化、景观化的现代公路交通网络和覆盖城乡的高速宽带信息网络，形成发达的交通、电力、通讯、水利等基础设施体系和配套完善的市政公共设施体系。生态环境得到改善，可持续发展能力不断增强，成为国内最适宜创业和生活的城市之一。

——基本形成与世贸组织规则接轨的市场经济体制。完成国有经济的战略性调整，大中型企业全面建立现代企业制度。形成比较完善的现代市场体系、社会保障体系、政策法规体系和公共行政服务体系，建立起更具活力、更加开放的经济体系。

——基本形成高度适应国内国际市场变化的开放格局。高新区和工业园区成为招商引资和出口创汇的主体，鞍钢与地方企业实现共兴共荣。营造出一流的投资环境，建立起没有地域和制度壁垒，人才、资本、信息、技术等生产要素在域内外之间、国有经济和非国有经济之间自由流动、合理配置的开放型发展模式。

——基本形成在国内外市场竞争中具有比较优势的现代工业体系。引进一批大项目，完成产业结构优化升级，形成以大企业、大集团为依托，以钢铁、纺织、食品加工等传统产业为支柱，以环保、新型材料两个新兴产业和电子信息等高科技产业为先导的现代制造业产业群，在世界产业分工中争得一席之地，构筑起现代工业强市的发展格局。

——基本形成区域性商贸旅游中心。建成业态先进、设施现代、功能完备的流通网络，构筑起大商业、大市场、大贸易的流通格局。开发培育出世界级旅游产品和旅游文化活动，建立起“游购娱吃住行”六大要素高度协调、与国际接轨的旅游接待服务体系，形成以优美环境和现代设施为依托，以观光旅游、休闲旅游、购物旅游、工业旅游、生态旅游为基本特征的旅游经济格局，成为国内外享有盛名的旅游目的地和中国北方旅游中心城市之一，打造出优秀旅游名市的靓丽名片。

——基本形成促进人的全面发展的先进文化体系。建成一批先进的文化设施，建立起发达的现代国民教育体系、科技创新体系和完善的医疗卫生体系，培养和引进一批国内外有影响的优秀人才，形成先进的文化体制、发达的文化产业和浓厚的文化氛围，满足人们日益增长的精神文化需求，树立起先进文化城市的崭新形象。

——基本形成分配合理、劳动致富的社会结构。建成公共就业服务体系，实现比较充分就业。工农差别、城乡差别、地区差别明显缩小，形成以技术工人、富裕农民、专业技术人员、个体工商户和行政与管理人员为主的中等收入阶层。到2010年，城市人均可支配收入达到13000元，农民人均纯收入达到6400元。

各位代表，上述目标是按照市委九届六次全会精神提出的，是鞍山全面建设小康社会的阶段性目标。经过这个阶段的建设，再奋斗几年，到2014年，提前六年实现国内生产总值翻两番，基本建成经济更加发展、民主更加健全、科教更加进步、文化更加繁荣、社会更加和谐、人民生活更加殷实的小康社会。到2020年，实现国内生产总值翻三番，使鞍山这座工业名城展现出勃勃生机和灿烂前景！

面对全市人民的热切期盼，新一届政府将全面贯彻落实党的十六大精神，高标准组织实施全面建设小康社会总体构想，做到发展有新思路，改革有新突破，开放有新局面，各项工作有新举措。

（一）把全面建设小康社会作为政府工作的行动纲领，加快推进工业化、信息化和现代化建设。坚持以信息化带动工业化，以工业化促进信息化，推进产业结构优化升级，走出一条具有鞍山特色的新型工业化道路。加快利用高新技术和先进适用技术改造传统产业。以鞍钢为主体，壮大钢铁深加工和机械制造产业群，成为国内最大的钢铁板材生产加工基地和配送中心。整合纺织行业资源，加快纺织工业园区建设，提高专业化协作水平，开发高档面料和自主服装品牌，成为国内著名的纺织品生产和销售集散地。大力开发绿色食品、保健食品和方便食品，成为辐射东北乃至全国的特色食品生产基地。积极培育高新技术产业，建设全国一流的高新技术产业开发区，建成环保、新型材料和软件产业基地。推进企业信息化，加快公共信息网络建设，打造数字鞍山。按照“二三一”顺序调整农村产业结构，用工业化理念谋划农业发展，用工业化方式组织农业生产，用工业化管理办好农事企业，用农村工业的增长带动农民收入的增加，使农村尽快走上工业化、产业化、城镇化的新路子。规划建成10个现代农业园区和10个农产品出口基地，培育20个省级以上龙头企业，“公司＋农户”带动面达到70%以上。大力发展非公有制经济和混合所有制经济，培育和扶持一批民营企业做大规模、做强产业、做响品牌。开发旅游大项目，深度整合周边旅游资源，开辟精品旅游线路，到2007年旅游综合收入占GDP比重达到9%。加快发展现代服务业，构筑多层次的社会化服务网络。

（二）把招商引资上项目作为经济工作主线，全面落实“一开三促”、建设开放型地区经济战略构想。坚持以项目为核心，做到内商外商一起招、内资外资一起引，通过大开放促进大发展。认真组织争取资金争取项目工作，不断加大固定资产投资力度。每年滚动推出投资亿元以上项目不少于100个，投资5亿元以上项目不少于20个，形成储备一批、推介一批、建设一批的项目工作格局。以国有企业嫁接改造、推进民营企业合资合作和引进独资大项目为重点，每年至少举办两次大型招商推介活动。采取园区招商、组团招商、高层推介等招商手段，提高招商引资成功率和实效性。积极调整出口产品结构，大力开拓国际市场，促进外贸出口持续快速增长，提高国民经济外向依存度。实施“走出去”战略，扩大对外交往，开展对外工程承包和劳务输出，鼓励有条件企业实施跨国经营。

（三）把扩大就业和完善社会保障体系作为政府“一号工程”，不断提高城乡居民生活水平。以高度的责任感和使命感，全力做好就业和社会保障工作。实施开发就业岗位五年攻坚行动，完成安置30万人就业目标。全面落实促进就业的各项政策，引导全社会转变就业观念。强化就业培训，提高劳动者就业技能。规范用工管理，维护劳动者合法权益。全面完善养老、失业、医疗保险制度和城市最低生活保障制度。依法扩大社会保险覆盖面，强化保费征缴，加大财政支出力度，广辟筹资渠道，不断增强社会保障能力。建成社会保障计算机信息网络，依托社区建立保障服务平台，提高社会化管理服务水平。鼓励和引导通过诚实劳动与合法经营致富，扩大中等收入阶层比重。实施东部山区开发工程，采取对口包扶措施，加大资金、技术和政策支持，用三至五年时间使全市贫困人口基本脱贫。

（四）把环境建设作为政府工作的切入点，将鞍山建设成为省内投资环境最好的地区。加快城市现代化建设，合理调整城市布局，拓展经营城市空间，完善城市基础设施和服务功能，增强城市的辐射力和带动力。加强以河流污染整治、空气污染治理和造林绿化为重点的城乡生态环境建设，争创“国家环保模范城市”和“国家园林城市”。全面推进县城、中心城镇和小城镇建设，不断提高城镇化水平。坚持不懈地实施软环境建设绿色行动，全面落实软环境建设的一系列政策规定，全力构筑适应市场经济发展、符合国际通行准则和WTO规则、外商和市民普遍满意的绿色服务通道。不断深化行政审批制度改革，完善公共行政服务体系，推行“阳光行政”。建立以服务对象为主体的外部评估制度，强化对机关行政效能和公务员行政行为的监督考核。采用下达过失单、强令整改等有效形式，坚决查处各类违规行为，实现在鞍山“办事不用求人、事事都有人管、说理必有地方”的理想目标。在全体市民中开展争做心胸开阔、热情好客、明礼诚信、助人为乐的“好市民”活动，让所有鞍山人和来鞍客人充分感受到鞍山美好家园的吸引力和宾至如归的感染力。

（五）把实现人的全面发展作为社会发展的总体目标，为鞍山现代化建设提供精神动力和智力支撑。立足于鞍山未来20年、50年甚至100年发展的要求，牢牢把握中国先进文化前进方向，坚持以人为本思想，弘扬和培育反映时代特征、具有民族特色的鞍山人精神，完善社会主义思想道德体系。加快建立和完善覆盖全社会的现代教育体系，建设全民学习、终身学习的学习型社会，培养和提高鞍山人的学习能力和创新能力。制定和实施地域文化发展规划，继承优秀历史文化，发展先进现代文化，提炼地域特色文化，升华鞍山人的个性文化，全面提升城市文化品位。培育壮大文体产业，建设和完善满足城乡不同层次人群需要的文化体育设施，建立起市场化运作、社会化参与的文化发展新机制。建立和完善卫生服务、医疗保健、疾病控制体系，提高医疗保健和人民健康水平。

（六）把开拓创新作为政府工作的主旋律，加快建设务实高效廉洁的服务型政府。用创新思维引领经济发展，冲破一切妨碍发展的思想禁锢，改革一切束缚发展的规定做法，消除一切阻碍发展的体制弊端，不害怕困难，不回避矛盾，不消极对待，深层次思考和规划鞍山的未来发展。赋予“谋划大事、自我加压、破解难题、深度推进”新的内涵，发挥主观能动性和创造性，敢于超越自我，虚心向先进地区学习，付出比别人更多的智慧，洒下比别人更多的汗水，瞄准国内一流水平赶超。深化行政管理体制改革，科学界定政府各部门职能，全面理顺政府与市场、政府与企业、政府与社会的关系。引入现代管理手段，加快“电子政府”建设。树立“以民为本、以客为尊”的政府服务理念，塑造具有人文关怀与亲和力的政府形象。

三、2003年经济和社会发展主要任务

今年是全面建设小康社会的起步之年，是新一届政府任期的第一年。全市经济和社会发展的总体要求是，坚持以项目为核心，以招商引资、发展民营经济和调整国有经济布局为重点，全面推进改革开放和现代化建设，实现经济总量、环境建设和人民生活水平再上一个新台阶。预期实现国内生产总值增长12%以上，完成全社会固定资产投资增长15%以上，地方财政一般预算收入增长12%以上，城市居民人均可支配收入增长8%，农民人均纯收入增长6%。在起步之年，新一届政府将牢记加速发展的政治责任，保持加速发展的精神状态，谋划加速发展的取胜之道，团结和带领全市人民，积极投身于鞍山改革开放和现代化建设，以更高的智慧，更大的手笔，创造新的业绩，铸就新的辉煌。

（一）抓住一批大项目，全力提升招商引资规模和水平，确保全年争取到位资金增长20%以上

完善项目滚动推出机制。市级项目库每季度调整一次，年内新规划超亿元项目50个，项目储备不少于100个。其中，超5亿元项目20个，超10亿元项目5个。确保有5个超5亿元项目取得实质性进展。

推进重点项目实施建设。建成H型钢、海诺二期工程等19个在建项目。开工建设台湾纺织工业园、30万吨大米及副产品深加工等33个大项目。完成年产30万吨杨木纸浆、30万吨不锈钢薄板等9个大项目前期工作。推动年产30万台移动通讯电脑等20个重大招商项目取得新的进展。

全面开展对外招商。发挥10个专业项目推进组及招商小分队作用，以台湾、香港、韩国、日本以及长江三角洲、珠江三角洲为重点招商地区，以跨国公司、世界500强和国内知名企业为重点招商对象，办好四月份香港和九月份北京招商推介会及海外学子创业周活动，争办台湾知名企业家辽东半岛观光周活动。年内引进5户以上国内外知名大企业。把扩大出口与利用外资放在同等重要的地位，支持更多的高新技术产品、机电产品以及资源深加工产品进入国际市场，确保全年出口创汇增长13%以上。

（二）深化国有企业改革，加快推进财政建设性国有资本的有效流动，确保资产经营收入6亿元

全面推广“两啤一化”模式。引进域内外资本、技术和管理，对20户国有大中型工商企业进行脱胎换骨式改造。重点推进美国欧文斯·科宁整体收购玻璃厂兴建PVC外墙挂板、挤塑保温板和玻纤瓦生产线；推进印尼武龙加鲁达收购一轧厂兴建30万吨H型钢生产线和沈阳昊怡重组鞍山一工等项目的实施；促进香港远东实业收购轧钢厂扩建30万吨汽车用钢生产线、美国七喜实业与华龙钢管合资生产20万吨大口径直缝焊管等签约项目的落实；推进市汽车配件厂、化建总公司、电子电力公司等与民营企业合资合作项目的对接。依法对15户劣势企业实施破产。规划鞍山工业园、高新技术产业园、钢铁工业区、鞍海新经济带和矿山带的“两园一区两带”工业布局。完成一百、天河等国有大中型商业企业对外合资合作。

调整国有经济布局。按照管资产与管人、管事相结合的原则，深化国有资产管理体制改革。对财政投入形成的国有资产，通过整体退出、转让股权、重组置换等方式，实现有效流动。重点推进天成市场国有资产拍卖，国际大酒店国有资产置换，国有苗圃出售，鞍信、合成国有股权出售，实现国有资产重组与流动6亿元。

全力支持鞍钢改革与调整。支持鞍钢加快冷轧2＃线、新1＃高炉、三炼钢异地改造

等重大项目建设，创建循环经济示范企业。协助做好钢绳生产线扩建、雾化铁粉生产线等10个重大项目的招商引资。支持鞍钢盘活地方企业闲置资产，推进冷弯型钢厂、北方铸钢厂、农用车制造总厂等与鞍钢合作项目的对接。在企业改制分流、社会保障、安置就业、经济适用房开发等方面提供优惠政策，创造宽松环境。

（三）坚持放心、放开、放手原则，大力发展民营经济，确保民营经济实缴税金增长15%以上

放心发展民营经济，就是不为姓“社”姓“资”问题所困扰。主动帮助民营企业实施超千万元项目128个，超亿元项目35个，做强做大宝德、西洋、后英、鞍轮等一批民营企业。扶持年产60万套全钢丝子午轮胎等6个超5亿元项目建设，帮助推进茧丝加工、滑石高性能系列母粒等10个超亿元项目建设。全市年销售收入超亿元民营企业增加到46个，超5亿元企业达到5个。培育西洋复合肥、心品二道茶饮品等10个省级以上知名品牌。

放开发展民营经济，就是国有经济可进入的领域都鼓励民营企业进入。要把发展民营经济与农业产业化相结合、与小城镇建设相结合、与国有企业改革相结合、与社区服务相给合，扶持舒妍芦荟、天天乳业等14家省市级农业产业化龙头企业，鼓励民营企业通过收购、兼并、控股等多种形式参与国有企业改革，引导民营企业进入社区开办便民利民服务业。

放手发展民营经济，就是要营造有利于民营经济发展的大环境。要完善民营企业激励机制，通过“以奖代补”和贷款贴息等办法，扶持“三十强”民营企业，奖励有突出贡献的民营企业家。建立民营企业创业服务中心和职工培训中心。鼓励发展为民营企业服务的人才、信息、法律、技术等各类中介组织。

（四）健全社会保障体系，努力扩大社会就业，确保全年新增就业岗位8万个

全面完成社会保障体系试点任务。依法扩大社会养老保险和医疗保险覆盖面，企业养老保险参保职工36万人，失业保险参保职工66万人，医疗保险参保人数71.2万人，保费收缴率达到95%。实现国有企业下岗职工向失业保险并轨3万人，全面偿还并轨人员内欠。建立依托街道社区的劳动保障服务平台，离退休人员全部纳入社区管理。建成市级社保计算机网络，医疗IC卡在参保人员中推广使用。

积极扩大社会就业。深入开展“双百”创业工程，发挥再创业服务中心的指导服务、创业示范和辐射带动作用。建立规范的公共就业服务体系和信息网络体系，提高用工信息的利用率和覆盖面。大力开展对下岗失业人员的免费职业介绍和再就业培训，探索“订单式培训”、“个性化培训”等新模式。全年实现就业培训2.5万人，就业援助8000人，城镇登记失业率控制在6%以下。

认真做好扶贫帮困工作。加快建立和完善救灾救助、社会救济、社会互助、社会福利服务四大体系。提高城镇居民最低生活保障管理与服务的社会化水平，保障对象月人均救助额由68元提高到80元，确保动态管理下的应保尽保。关心老干部、老模范、老复员军人和社会弱势群体的生活，保障其基本生活需要。整合社会力量，调整和完善扶贫帮困体系，包户扶持脱贫率达到50%，解决贫困户住房400户。

加强社区建设。以争创全国社区建设示范城为目标，在全市80%街道建立面积在500平方米以上、具有多种服务功能的服务中心，90%的社区“一站四室”达到省规定标准。做大做强社区“四大公司”。在老社区开展物业管理试点工作。

（五）加快发展高新技术产业，加速推进高新区建设，确保高新技术产品产值增长30%以上

加快运用高新技术提升传统产业。发挥鞍山与上海交大制造业数字化联合技术中心作用，推进金堡、亨通等15家示范企业信息化进程。依托天津电传所、德国西门子和美国ABB等技术优势，整合华冶、中新、立德、宏源和荣信等电控设备加工企业，建设电控设备产业基地。推进广电集团与清华同方合作，建设全国最大的数字广播电视发射设备加工基地。

大力推进高新技术产业化。以鞍山清华紫光环保设备有限公司为龙头，建设环保产业园。以科大聚龙为主体，推进捆钞机、清分机等金融机具产业化进程，建设全国最大的金融设备加工基地。以沈阳化工研究院和沈阳科大为依托，吸纳惠丰等中小化工企业，建设精细化工产业园。开展南果梨常温储存、纳米粉体材料等10个重点课题技术攻关。鼓励发展科技中介服务机构，推进民间资本与科技项目对接，做大做强聚龙、森远等30户民营高科技企业。全年完成研发和科技成果转化100项。

创新高新区发展模式。与国内外大企业合资合作，组建高新区开发建设公司，推进基础设施建设市场化运作。开工建设高新大厦等项目，建成清华博士生实践基地，全年完成基础设施投资3亿元。规划建设鞍钢附企工业园、汽车配件园、食品工业园、农机工业园和绿色环保动力科技园，形成“一区多园”发展模式。重点推进台塑集团舒美家具板和BOPP膜、微晶玉石装饰材料、托普软件园三期工程等项目的前期工作和开工建设。引进投资5亿元以上项目2个，亿元以上项目10个，新增进区企业100家，新孵化企业60家。全年高新技术产品产值、财政收入、实际利用外资调入额和出口创汇等主要指标均比上年增长30%以上。

（六）强化农业基础地位，加快农业产业化和农村城镇化进程，确保农民人均纯收入超过4000元

深化农村各项改革。按照中央和省统一部署，积极稳妥地推进农村税费改革，从根本上解决对农民乱收费问题，保护农民合法权益，确保农民负担减轻，确保农村基层组织正常运转，确保义务教育经费。强化农业服务体系建设，健全农产品标准体系、认证体系和检测体系，提高农产品市场竞争力。

推进农业产业化经营。按照“二三一”产业发展思路，调整农村产业结构，增加县乡财政收入，壮大县域经济实力。推广“公司+农户”经营模式，龙头企业带农户比例达到50%。发展50万亩优质水稻和45万亩无公害蔬菜。壮大花卉、食用菌等十大产业化基地，培育奶牛、桑蚕、芦荟、山药等四个新兴产业化基地，做大做强南果梨等5大名牌产品。完成台安山药深加工、宁远蔬菜批发市场改造等项目建设，开工建设完达山4万吨乳制品、岫岩板栗深加工项目，做好50万亩杨树纸浆林等项目的规划论证。加快推进千山宁远、海城王家堡子等4个省级和6个市级现代农业园区建设，年内每个乡镇至少规划建设一个现代农业园区。

加快农村城镇化进程。有重点、分层次、按步骤地推进小城镇和村屯建设。以工业园区、专业市场为重点，高水平搞好小城镇建设规划，把“经营城市”理念引入小城镇建设，完善基础设施，发展特色产业。改革小城镇户籍管理制度，鼓励和引导农村劳动力到城镇从事二、三产业。全面加强农村环境整治，重点搞好植树造林和绿化美化，年内建设宽500米、长5.7公里环城绿化屏障,完成沈盘路、鞍海路、鞍羊路、大盘路两侧绿化,全市造林面积达到30万亩。新建蓄水、供水、节水工程500处，完成水土流失治理30万亩，基本解决农村人畜饮水困难问题。努力增加农民收入。启动东部山区开发工程，加大西部沿河扶贫开发力度，支持少数民族地区发展，从根本上解决上述地区农民收入偏低问题，促进均衡发展。全年为农村劳动力提供就业岗位2万个，发展个体私营业户5000个，新增劳务输出6000人。帮扶43个贫困村、4万贫困户实现脱贫致富，农村贫困户人均收入增加200元以上。

（七）着力提升旅游业，积极发展现代服务业，确保第三产业增加值增长14%以上

积极争创中国最佳旅游城市。编制鞍山旅游业发展总体规划，制定重点景区专项规划。启动汤岗子分时度假区、玉佛苑二期工程、乐雪绿色生态观光园、高尔夫球训练场等旅游项目。推进岫岩玉雕博物馆、二一九公园改造、北方梅园等项目建设。完成汤岗子温泉度假区、宁远农牧业主题公园等项目规划。做强叫响五大旅游品牌，把“鞍钢工业游”建成国家级工业旅游示范点。整合周边旅游资源，精心设计以鞍山为轴心的旅游线路，挖掘旅游产品的文化内涵，开发特色旅游项目和旅游商品。强化旅游宣传促销活动，拓展国内外客源市场。进一步提高节庆活动的商业化运作水平，办好第四届中国·鞍山千山国际旅游节。全年实现旅游综合收入48亿元，比上年增长23%以上。

加快发展现代服务业。实施商业发展规划，利用现代流通业态改造传统商业，大力发展连锁经营、物流配送、电子商务。加快推进中海物流鞍山配送中心、大福源超市等重点项目建设。积极发展房地产、金融、保险、会计、咨询等现代服务业。整顿和规范市场经济秩序，健全适应市场经济的社会信用体系。

（八）深度推进“经营城市”，进一步完善城市功能，确保全年完成城市建设投资20亿元

拓宽城建筹资渠道。对经营性土地实行招标拍卖或挂牌出让，推进公益性国有资产的有效流动和国有非住宅房产的有偿使用，对到期的1200台出租车经营权实行公开拍卖，出售路桥等市政设施冠名权，全年筹集建设资金15亿元。

适时启动鞍山新区建设。按照基本形成现代大都市城市框架的远景目标，以在汤岗子一带规划建设环境优美、设施齐全、生态良好的鞍山新区为切入点，全面规划建设鞍海新经济带。以商业化运作为手段，通过土地质押筹措启动资金，通过招商引资实施项目建设。通过新区和鞍海新经济带的开发，打开城市发展空间，拓展经营城市领域，提高城市化水平，提升城市的辐射力、带动力和综合实力。

加强城乡基础设施建设。积极推进引汤入鞍扩建、节能环保型城市集中供热、西部第二污水处理厂二期工程、有轨电车改造四个打捆项目建设。完成垃圾处理厂二期工程。大修改造人民路、民生路、健身街等六条道路的部分路段，基本完成城市主干道改造任务。打造鞍山商务中心，按照集旅游、购物、休闲于一体的要求，高水平规划站前商业街改造工程。建成城市规划展示厅，成为市民参与城市规划建设和实施规划监督的窗口。建成国际会议中心。完成站前客运站和火车站内部、站台、出口的改造。全面开展城市建筑、道路、社区地名的清理整顿，完成50%设标任务。

加强城市环境综合治理。实施千山景观路规划建设，拆迁七号桥至千山正门两侧的陈旧建筑，并进行高水平绿化，建成千山路两端生活服务区。新植15条道路行道树，栽植大树5万株，新建草地20块，新增绿地9万平方米。启动主干道拆墙透绿三年规划，落实门前绿化“四包”责任制，开展认养绿地和树木活动。专项整治占道经营，城市27条主干道及站前地区全面达标。完成200万平方米市属居民小区改造任务，协调鞍钢等中省直单位启动所管小区改造工程。整治主街主路居民阳台和停车场，规范户外广告牌匾和路牌街牌，粉饰临街建筑100万平方米。全面启动城市供暖收费体制改革。加强城市防洪体系建设，对杨柳河、南沙河进行综合整治。通过市场化运作，全面提升公厕档次。治理机动车尾气，控制二次扬尘，全面治理低空污染。推进矿山生态恢复工作，完成鞍钢炼铁厂一号高炉改造等15项重点环境治理工程，巩固国家卫生城市创建成果。

（九）加强财税金融工作，构筑公共财政体制框架，确保地方可支配财力突破50亿元

推进财政体制改革。适时出台分税制财政体制改革方案，调整税源、税率分享比例，合理分配市、县（区）财政资源，建立与分税制财政体制相适应的转移支付制度。改革财政预算编制办法，全面实行部门预算，建立统一完整的政府收支预算体系。

做好增收节支工作。坚持依法治税，全面清理现行的各项税收优惠政策。加大税收征管力度，规范非税收入管理，加强对高收入者、个体工商户、私营企业以及各类改组、改制企业的税收征管，开展对重点行业的税收专项整治，严厉打击“偷、逃、骗、抗”税行为，把该收的钱一分不少地收上来。推行国库集中收付制度，扩大政府采购范围，提高财政资金的使用效率。优化财政支出结构，大力压缩一般性开支，保证公教人员工资，保证社会保障支出，保证各项法定支出。树立艰苦奋斗的思想，遵循勤俭节约、艰苦创业的原则，量力而行，精打细算，不该花的钱一分也不能花。

建设金融安全区。强化对“两债”兑付工作的监控协调，健全和完善金融风险预警机制。落实减债偿债责任，大力清收金融债权，压缩不良贷款比例，提高金融运行质量。加大金融执法、监督力度，形成能借能还的良性循环信用机制。

（十）加强社会主义民主与法制建设，促进社会全面进步，树立高效务实廉洁的政府形象

完善社会主义民主政治。坚持重大事项向人大及其常委会报告制度，自觉接受人大监督。积极支持政协履行政治协商、民主监督、参政议政职能，加强同各民主党派、工商联和无党派爱国人士的联系，充分发挥工会、共青团、妇联等群团组织作用。完善基层民主选举制度，推进社区居民自治。

加强社会主义法制建设。深入开展“四五”普法教育，提高全民法律意识，提高公职人员法律素质。加大行政立法工作力度，规范法律服务。严厉打击各种刑事犯罪和经济犯罪，防范和惩治邪教组织的犯罪活动，建立对暴力恐怖事件和重大突发事件的处置机制，做好人民内部矛盾纠纷排查调处工作，把综合治理的各项措施落实到基层，建立起长效机制。加强安全生产和安全防火工作，全力维护社会稳定。

加快先进文化城市建设。巩固和提高创建全国文明城市成果，大力倡导公民基本道德规范，加强社会公德、职业道德和家庭美德教育，深入开展群众性精神文明创建活动，提高市民文明素质和城乡文明程度，打造诚信鞍山。深度推进专业表演艺术团体改革，组建演艺和文物经营公司，建成市博物馆和戏曲剧院综合艺术楼，举办首届鞍山文化艺术节。全面实施中小学校规范化达标工程，推行城区小学小班化授课和双语教学试点。在城区普及高中阶段教育，义务教育入学率保持在98%以上，初中辍学率降至3%以下。建设一中新校园，确保鞍山师范学院通过教育部达标验收。加强师德教育，着力解决教育热点问题。全年吸引外国专家、海外留学人员和国内高层次人才100人次以上，引进大学本科以上人才1200人以上。调整优化卫生资源，城市社区卫生服务机构设置达100%，可服务社区人口达100%。深化农村医疗卫生体制改革，着力改善农村卫生状况。建成市体育中心网球馆和综合训练馆，完善全民健身服务网络，提高竞技体育水平。进一步做好计划生育、广播电视、双拥、民族、宗教、侨务、人防、妇女、儿童、老龄、史志、档案、民兵预备役、气象、地震等方面工作，促进经济和社会协调发展。

加强政府自身建设。牢固树立以民为本、全心全意为人民服务的思想，身体力行“三个代表”重要思想，常怀爱民之心，常思富民之策，常兴为民之举，努力创造更加优异的工作业绩。坚决克服官僚主义和形式主义，经常深入基层，倾听群众呼声，办实事、办好事。遵循WTO规则，继续推进政府职能转变，全面落实软环境建设绿色行动一系列规章制度，深入开展纳税人评议行风活动，让群众、让投资者感到办事方便和满意。强化公务员行为规范教育，全面推行竞争上岗和岗位轮换，提高公务员队伍的整体素质。认真落实廉政建设责任制，建立和完善领导干部离任审计和重点工程、专项资金使用情况审计制度，坚决查处吃拿卡要、索贿受贿、贪污腐化等违法违纪行为，纠正行业不正之风。

各位代表，我们已经进入全面建设小康社会的新的历史时期。面对新的征程、新的机遇、新的挑战，让我们高举邓小平理论伟大旗帜，全面实践“三个代表”重要思想，在以胡锦涛同志为总书记的党中央坚强领导下，与时俱进，开拓创新，为共同创造我们的幸福生活和美好未来而努力奋斗！
